# Cardiac surgery patient: differentiating targets for psychotherapy

**DOI:** 10.1192/j.eurpsy.2022.1915

**Published:** 2022-09-01

**Authors:** O. Nikolaeva, T. Karavaeva, E. Nikolaev, A. Zakharova, G. Dulina, D. Hartfelder

**Affiliations:** 1 Ulianov Chuvash State University, Department Of Faculty And Hospital Therapy, Cheboksary, Russian Federation; 2 Republican Cardiology Clinic, Cardiosurgery Unit, Cheboksary, Russian Federation; 3 St. Petersburg State University, Department Of Medical Psychology And Psychophysiology, St. Petersburg, Russian Federation; 4 V. M. Bekhterev National Medical Research Center for Psychiatry and Neurology, Department For Treatment Of Borderline Mental Disorders And Psychotherapy, St. Petersburg, Russian Federation; 5 St. Petersburg State Pediatric Medical University, Department Of General And Applied Psychology With A Course In Biomedical Disciplines, St. Petersburg, Russian Federation; 6 N.N. Petrov National Medical Research Center of Oncology, Scientific Department Of Innovative Methods Of Therapeutic Oncology And Rehabilitation, St. Petersburg, Russian Federation; 7 Ulianov Chuvash State University, Social And Clinical Psychology Department, Cheboksary, Russian Federation

**Keywords:** cardiac surgery patients, psychotherapeutic targets, levels of psychotherapeutic targets

## Abstract

**Introduction:**

Differentiation of targets for psychotherapy allows determining certain ways and priorities in psychological treatment of a patient.

**Objectives:**

To work out a multi-level system of psychotherapeutic targets for clinical groups of cardiac surgery patients (CSPs).

**Methods:**

Clinical and psychological analysis of 152 CSPs who were to undergo different types of cardiac surgery treatment.

**Results:**

We have established four levels of psychotherapeutic targets: a patient’s response to surgery, psychopathologic manifestations, personality’s dysfunctional characteristics, and social interaction specificities. Towards CSPs with open-heart coronary artery bypass grafting, the targets appeared to be as follows: low expectations from surgery, low hopes for recovery, low level of satisfaction with life, depressive disorders with somatic manifestations, cognitive abnormalities, anxiety manifestations, manifestation of hostility, rejection of the past, inclination for fatality, reduced vitality, reduced social activity, expectation of help from closest people.Towards CSPs indicated to open-heart aortic valve repair surgery, psychotherapeutic targets were as follows: high expectations from surgery; moderate fear of death; not feeling well; low spirits; depressive disorders with somatic and cognitive-and-affective manifestations; cognitive abnormalities; anxiety manifestations; manifestation of hostility; rejection of the past; reduced hedonism; expectation of help from closest people; reduced social activity. Towards CSPs indicated to minimally invasive surgery, we set such targets as: moderate expectations from surgery; apparent fear of death; depressive disorders with somatic manifestations; anxiety manifestations; cognitive abnormalities; rejection of the past; expectation of help from closest people; reduced social activity.

**Conclusions:**

Psychotherapy of CSPs that includes the established targets can contribute to personalized approach in a patient’s treatment.

**Disclosure:**

No significant relationships.

